# Diversity and spatiotemporal dynamics of fungal communities in the rhizosphere soil of *Lycium barbarum* L.: a new insight into the mechanism of geoherb formation

**DOI:** 10.1007/s00203-022-02781-5

**Published:** 2022-02-26

**Authors:** Yuekun Li, Kaili Chen, Siyang Liu, Xiaojie Liang, Yajun Wang, Xuan Zhou, Yue Yin, Youlong Cao, Wei An, Ken Qin, Yanfei Sun

**Affiliations:** 1 National Wolfberry Engineering Research Center, Wolfberry Science Research Institute, Ningxia Academy of Agriculture and Forestry Sciences, Yinchuan, 750002 China; 2 The College of Life Sciences, Shihezi University, Shihezi, 832003 China

**Keywords:** *Lycium barbarum* L., Geoherbs, Rhizosphere soil, Fungal community diversity

## Abstract

*Lycium barbarum* L. is a well-known traditional geoherb in Ningxia, China. The fruits of *L. barbarum* contain several dietary constituents, and thus, they exert many beneficial effects on human health. However, a few studies have been conducted on the geoherb *L. barbarum* and its rhizosphere soil fungal community. In this study, we determined the physicochemical properties and fungal community structure of rhizosphere soil of *L. barbarum* from three regions of China, namely Ningxia (NX), Qinghai (QH), and Xinjiang (XJ), during three development stages of *L. barbarum*. Soil pH varied between 7.56 and 8.60 across the three regions, indicating that alkaline soil is conducive to the growth of *L. barbarum*. The majority of soil properties in NX, an authentic geoherb-producing area, were substantially inferior to those in XJ and QH during all three developmental stages. Total sugar, polysaccharide (LBP), and flavonoid contents were the highest in wolfberry fruits from NX. High-throughput sequencing showed that the abundance of the soil fungal population in NX was higher than that in QH and XJ during the flowering and fruiting stage and summer dormant stage. Moreover, the soil fungal diversity increased with the development of wolfberry. *Ascomycota* and *Mortierellomycota* were the predominant phyla in the rhizosphere fungal communities in all samples. Redundancy analysis showed a significant correlation of the soil-available phosphorus and LBP of wolfberry fruits with the fungal community composition. The characteristics of rhizosphere fungal communities determined in the present study provide insights into the mechanism of geoherb formation in NX wolfberry.

**Supplementary Information:**

The online version contains supplementary material available at 10.1007/s00203-022-02781-5.

## Introduction

*Lycium barbarum* L. (wolfberry), belonging to the family Solanaceae, is one of the most important traditional medicinal plants that is widely cultivated in Ningxia and Qinghai Provinces in northwest China. The utilisation of *L. barbarum* fruits has increased gradually over the past 2 decades because of their proven nourishing value, anti-inflammatory and antiageing effects, and crucial role in the prevention and treatment of various chronic diseases (Shi et al. 2017; Cenariu et al. 2021). Wolfberry contains many nutrients with high biological activity, and its extracts exhibit immunomodulation and antitumour activities (Zhang et al. 2011; Huang et al. 2012; Kulczyński and Gramza-Michałowska 2016). Several studies have reported that *L. barbarum* polysaccharides (LBPs), a major active ingredient extracted from the fruits, possess a remarkable immuno-modulatory activity (Amagase and Farnsworth 2011; Cheng et al. 2015). In addition, phytochemical studies have revealed the presence of numerous secondary metabolites such as phenolic amides (Kai et al. 2015; Pei-Feng et al. 2017), alkaloids (Kun et al. 2011), peptides (Morita et al. 1996), and flavonoids (Qian et al. 2003) in *L. barbarum*. The clinical efficacy of wolfberry has not been established yet, although its pharmaceutical properties have been determined in in vitro and in vivo studies, suggesting that it may be beneficial in the prevention and treatment of tumours (Potterat 2010). *Lycium barbarum* fruits also exert antitumour effects, which can be mainly attributed to the presence of carotenoids (Hsu et al. 2017).

Wolfberry in Ningxia Province has emerged as a well-known geoherb because of the unique geographical environment and climatic conditions in this province. Presently, the research on *L. barbarum* is mainly focussed on the cultivation, breeding, salt tolerance mechanism, effect of soil salt content on the accumulation of polysaccharide, chemical composition analysis, and pharmacological analysis (Masci et al. 2018; Zhang et al. 2019; Zhang et al. 2020; Yu et al. 2020b). However, only a few studies have investigated the quality of Ningxia wolfberry and soil microbial communities. In the plant rhizosphere micro-ecosystem, plants release various root exudates under the influence of different environmental factors, which creates a unique and favourable environment for microbial growth and activity (Bais et al. 2006). Nitrogen-fixing rhizobia and mycorrhizal fungi in the rhizosphere have been reported to greatly influence the plant nutrient status (Mendes et al. 2013; Lu et al. 2018). Rhizosphere fungi are closely linked to plant health and growth due to their roles in antagonising pathogens, decomposing plant residues, and providing nutrients. Rhizosphere fungi can not only promote the absorption of soil nutrients by plants but also improve the yield and quality of wolfberry. Therefore, studying *L. barbarum* and its rhizosphere fungi can be useful for understanding the mechanism through which rhizosphere fungi increase the yield and nutritional value of the fruit.

Abundant microbes are present in the rhizosphere; however, only 0.1–1% of the environmental microorganisms can be cultivated by traditional methods, which cannot fully reflect the real situation of the environmental microbial community. High-throughput sequencing technology has been widely used for studying the microbial community structure due to the advantages of high-throughput, low price, and short operation cycle (Ying et al. 2012; Zuo et al. 2021). Therefore, in the present study, we characterised the rhizosphere fungal community of wolfberry in different regions during three stages, namely the sprouting stage, flowering and fruiting stage, and summer dormant stage. In addition, we analysed the environmental factors and fungal community composition related to the main nutrients of wolfberry to investigate the correlation between Ningxia wolfberry quality and soil microbial flora from the perspective of rhizosphere fungal community. We believe that the present study could provide new perspectives to explore geoherbs and guide Ningxia wolfberry production practices.

## Materials and methods

### Wolfberry material and soil sampling

Similar wolfberry cuttings were artificially cultivated in Jinghe county (Xinjiang Province, XJ), Nuomuhong county (Qinghai Province, QH), and Zhongning county (Ningxia Province, NX), China, and were managed uniformly in an experimental base. We sampled the soil surrounding the plant roots and randomly collected five soil cores from each plot to form a composite sample for each soil sample. The rhizosphere soil samples were collected at three stages, namely sprouting stage (SS), flowering and fruiting stage (FFS), and summer dormant stage (SDS), from the same plant in 2019. The soil samples were placed in sterile sealed plastic bags and then stored in two parts: one part was stored at − 80 °C for microbial diversity detection, whereas the other part was air dried and stored at room temperature for the determination of soil physical and chemical properties. Three biological replicates of each variant were performed. Sample information is presented in Table S1. The soil samples were collected from the surrounding of the plants from which wolfberry fruits were collected; then, the ripe fruits were collected, dried, mixed, and stored in bags at room temperature.

### Soil properties and the main effective components of wolfberry

The soil physical and chemical characteristics were analysed according to a procedure described previously (Ehrmann and Ritz 2014; Wang et al. 2020). The soil water content was measured after oven drying the soil for 6 h at 105 °C. The soil pH was measured in a 1:2.5 (wt/vol) mixture of soil and water using a pH metre; the soil samples were ground to measure total nitrogen (TN) using a CHNOS elemental analyzer (Wang et al. 2020). The soil organic carbon (SOC) was measured using a method described in a study (Zhao et al. 2014). Soil total phosphorus (TP) was measured through flame photometry. Soil total potassium (TK) was measured through alkali fusion-Mo-Sb Anti spectrophotometry; soil-available nitrogen (AN) was measured using a dual-wavelength scheme with an UV spectrophotometer (Kelly and Love 2007); soil-available potassium (AK) was measured using a flame photometer, and available phosphorus (AP) was measured using the molybdenum blue method (Crouch and Malmstadt 1967). The electrical conductivity (EC) of the soil samples was measured in a 1:5 (wt/vol) mixture of moist soil and boiled water using a conductivity metre. The contents of total sugar, polysaccharide, betaine, flavonoids, and carotene in the fruits were determined using a spectrophotometer with NY/T1676-2008, NY/T1746-2009 and GB/T5009.83-2003.

### DNA extraction, PCR amplification, and MiSeq

Total DNA was extracted using the genome extraction kit (Powersoil^®^ DNA isolation kit) following the manufacturer’s protocol. The concentration of the extracted DNA was determined on the NanoDrop One system (NanoDrop Technologies, Wilmington, DE, USA), and the DNA degradation degree was examined through 1% agarose gel electrophoresis. Then, the ITS region of the fungal internal transcribed spacer (ITS) was amplified using the ITS1F forward primer (5′-CTTGGTCATTTAGAGGAAGTAA-3′) and ITS2 reverse primer (5′-GCTGCGTTCTTCATCGATGC-3′), and the PCR products were sequenced through paired-end sequencing on the Illumina (MiSeq) platform. The raw sequences were deposited into the National Centre for Biotechnology Information with SUB10211634 in PRJNA759085.

### Analysis of the high-throughput sequencing data

Bioinformatic analyses were performed using QIIME (Quantitative Insights into Microbial Ecology, version 2.0) according to the methods described in a study (Caporaso et al. 2010; Sun et al. 2018). Briefly, the adapter sequences and low-quality sequence were discarded, and the clean data were clustered into operational taxonomic units (OTUs) at 97% similarity (Edgar 2010). Alpha diversity was estimated in QIIME based on the OTU results. Taxonomy was assigned using the RDP Classifier against the Unite (Release 8.2 http://unite.ut.ee/index.php) for fungal OTUs (Wang et al. 2007).

### Statistical analysis

The significance of differences in soil properties between the treatments was determined using the Duncan’s test at a 95% confidence level in SPSS 20. 0. The relationship among the soil properties, contents of effective medicinal components of wolfberry, and soil rhizosphere fungal community was determined through a canonical redundancy analysis (RDA) in CANOCO software version 4.5. A *P* value of < 0.05 was considered to denote the significance threshold in all tests.

## Results

### Analysis of rhizosphere soil properties and nutritional ingredients of wolfberry

The rhizosphere soil of *L. barbarum* L. was collected from Xinjiang (XJ), Qinghai (QH), and Ningxia (NX) Provinces, China, and the physicochemical characteristics of rhizosphere soils were determined during the SS, FFS, and SDS (Table 1, Table S2). Most soil properties in NX were different from those in XJ and QH. The EC and contents of SOC, TN, TP, AN, AP, and AK in rhizosphere soil from NX were significantly lower than those in the soil samples from XJ and QH during the three developmental stages. The rhizosphere soil of *L. barbarum* was alkaline; the pH was not significantly different between the samples from QH and XJ; however, the soil from NX had the highest pH during the three developmental stages. The AN, AP, and AK contents exhibited significant differences in the three developmental stages. The SOC, AN, and AP contents were the highest in soil samples from QH, whereas the AK content was the highest in the XJ samples during the FFS.

**Table 1:** Basic physicochemical properties of rhizosphere soil samples from three regions during three developmental stages

	Sprouting stage			Flowering and fruiting stage			Summer dormant stage		
	NX	QH	XJ	NX	QH	XJ	NX	QH	XJ
pH	8.60 ± 0.04^a^	8.16 ± 0.01^b^	8.19 ± 0.01^b^	8.43 ± 0.03^a^	7.83 ± 0.01^b^	7.56 ± 0.03^b^	8.52 ± 0.02^a^	8.01 ± 0.01^b^	8.02 ± 0.01^b^
EC (mS/cm)	0.068 ± 0.01^b^	0.127 ± 0.01^a^	0.136 ± 0.00^a^	0.046 ± 0.00^c^	0.099 ± 0.00^b^	0.383 ± 0.00^a^	0.065 ± 0.00^c^	0.163 ± 0.01^a^	0.149 ± 0.01^b^
SOC (g/kg)	2.82 ± 0.09^c^	16.70 ± 0.20^a^	12.90 ± 0.15^b^	3.82 ± 0.32^c^	18.80 ± 0.06^a^	11.80 ± 0.36^b^	3.67 ± 0.15^c^	14.50 ± 0.23^a^	11.90 ± 0.10^b^
TN (g/kg)	0.31 ± 0.02^c^	1.05 ± 0.02^a^	0.80 ± 0.01^b^	0.25 ± 0.01^c^	1.31 ± 0.03^a^	0.81 ± 0.02^b^	0.28 ± 0.01^c^	0.87 ± 0.00^a^	0.73 ± 0.00^b^
TP (g/kg)	0.93 ± 0.01^c^	1.87 ± 0.02^a^	1.37 ± 0.02^b^	0.48 ± 0.01^c^	1.18 ± 0.03^a^	0.79 ± 0.01^b^	0.45 ± 0.01^c^	1.06 ± 0.01^a^	0.81 ± 0.00^b^
TK (g/kg)	18.90 ± 0.12^b^	19.10 ± 0.11^b^	23.30 ± 0.17^a^	18.10 ± 0.12^c^	20.10 ± 0.23^b^	21.90 ± 0.12^a^	15.90 ± 0.12^c^	17.30 ± 0.29^b^	21.70 ± 2.89^a^
AN (mg/kg)	18.00 ± 0.73^c^	89.00 ± 4.04^b^	97.00 ± 2.51^a^	127.00 ± 4.04^b^	221.00 ± 2.08^a^	123.00 ± 5.50^b^	10.00 ± 1.00^c^	66.00 ± 1.53^a^	55.00 ± 0.58^b^
AP (mg/kg)	11.70 ± 0.38^c^	164.00 ± 5.86^a^	32.00 ± 0.53^b^	39.40 ± 1.76^b^	176.00 ± 4.58^a^	26.10 ± 0.31^c^	23.00 ± 0.20^b^	70.10 ± 3.84^a^	16.80 ± 0.25^c^
AK (mg/kg)	80.00 ± 0.00^c^	168.00 ± 2.89^a^	152.00 ± 2.89^b^	98.00 ± 2.89^c^	203.00 ± 2.89^a^	207.00 ± 2.89^a^	75.00 ± 0.00^c^	125.00 ± 5.00^b^	143.00 ± 2.89^a^

^a^^,b,c^Values represent means ± standard deviations (SDs) (n = 3). Values within a row followed by different lowercase letters are significantly different (P < 0.05, Duncan’s test)

*EC* electrical conductivity; *SOC* soil organic carbon; *TN* total nitrogen; *TP* total phosphorus; *TK* total potassium; *AN* available nitrogen; *AP* available phosphorus; *AK* available potassium

### Analysis of nutritional ingredients of wolfberry

Then, the main active ingredients of *L. barbarum* fruits were quantified in the samples from NX, XJ, and QH (Table 2, Table S3). The highest total sugar, LBP, and flavonoid contents were detected in the fruits from NX. The betaine and carotenoid contents were significantly higher in the fruits from QH than in those from NX and XJ. Thus, the quality of NX wolfberry was superior to those of the fruits from other two regions because it contained the largest amount of active ingredients.

**Table 2:** Content analysis of the main active ingredients of *L. barbarum*

Sample	Total sugar/(g/100 g)	LBP/(g/100 g)	Betaine/(g/100 g)	Flavonoids/(g/100 g)	Carotenoid/(g/100 g)
NX	48.58 ± 0.48^a^	2.70 ± 0.02^a^	0.82 ± 0.04^b^	0.15 ± 0.02^a^	0.37 ± 0.01^b^
QH	46.18 ± 0.31^c^	1.84 ± 0.18^b^	0.94 ± 0.06^a^	0.11 ± 0.01^b^	0.42 ± 0.01^a^
XJ	47.36 ± 0.15^b^	2.72 ± 0.18^a^	0.86 ± 0.04^ab^	0.13 ± 0.01^ab^	0.24 ± 0.01^c^

^a^^,b,c^Values represent means ± standard deviations (SDs) (n = 3). Values within a row followed by different lowercase letters are significantly different (P < 0.05, Duncan’s test)

*LBP* polysaccharide

### α-Diversity of rhizosphere soil fungal communities

The fungal communities were characterised through next-generation sequencing of nuclear ribosomal ITS1 in 27 soil samples. A total of 1,948,798 high-quality reads were obtained from all samples (Table S4). The high-quality reads were clustered into 1762 microbial OTUs at 97% similarity after removing the OTUs that were unassigned or not assigned to the target species (Table S5). To compare fungal diversity, we compared the rarefaction curves. Species richness is represented in these rarefaction curves and was measured according to the number of OTUs using a cutoff of 97% for similarity in sequence. The majority of the rhizosphere soil samples saturated 300–500 OTUs for fungi (Fig. S1A).

An analysis of α-diversity indicated that the Sobs, Chao, Shannon, Simpson, and ACE indices were higher in NX rhizosphere fungal communities than in QH and XJ rhizosphere fungal communities during the FFS and SDS (Table 3). In the SS, α-diversity indices were lower in the rhizosphere soil from NX than in those from QH and XJ. Moreover, the fungal alpha diversity showed an increasing trend with the growth of *L*. *barbarum*, suggesting that the fungal community abundance was gradually increased in rhizosphere soil (Table 3).

**Table 3:** Alpha diversity indices of the rhizosphere soil fungi communities

Sample	OTUs	ACE	Chao1	Shannon	Simpson	Sobs
NX-SS	322.67 ± 10.60	351.11 ± 17.06	352.88 ± 12.66	3.52 ± 0.07	0.06 ± 0.01	324.33 ± 13.50
NX-FFS	464.00 ± 26.66	506.86 ± 27.74	499.75 ± 26.47	2.94 ± 0.06	0.15 ± 0.01	463.33 ± 23.46
NX-SDS	470.00 ± 53.33	532.39 ± 47.39	520.19 ± 53.81	2.88 ± 0.66	0.20 ± 0.12	470.67 ± 52.20
QH-SS	360.33 ± 19.04	434.65 ± 35.53	429.23 ± 31.74	3.39 ± 0.10	0.07 ± 0.01	360.00 ± 18.03
QH-FFS	261.33 ± 91.25	318.08 ± 101.09	322.95 ± 100.12	2.66 ± 0.98	0.16 ± 0.16	260.33 ± 89.76
QH-SDS	356.67 ± 7.57	402.65 ± 8.33	398.86 ± 11.35	3.77 ± 0.12	0.04 ± 0.01	355.33 ± 9.07
XJ-SS	365.67 ± 16.26	435.52 ± 33.60	436.69 ± 34.64	2.74 ± 0.18	0.19 ± 0.05	369.33 ± 17.10
XJ-FFS	413.00 ± 42.23	482.80 ± 45.74	472.99 ± 49.18	2.73 ± 0.16	0.19 ± 0.03	411.67 ± 39.83
XJ-SDS	455.33 ± 87.56	509.69 ± 72.07	511.17 ± 73.14	3.33 ± 0.74	0.11 ± 0.09	453.67 ± 85.33

### Fungal β-diversity

**Fig. 1:**
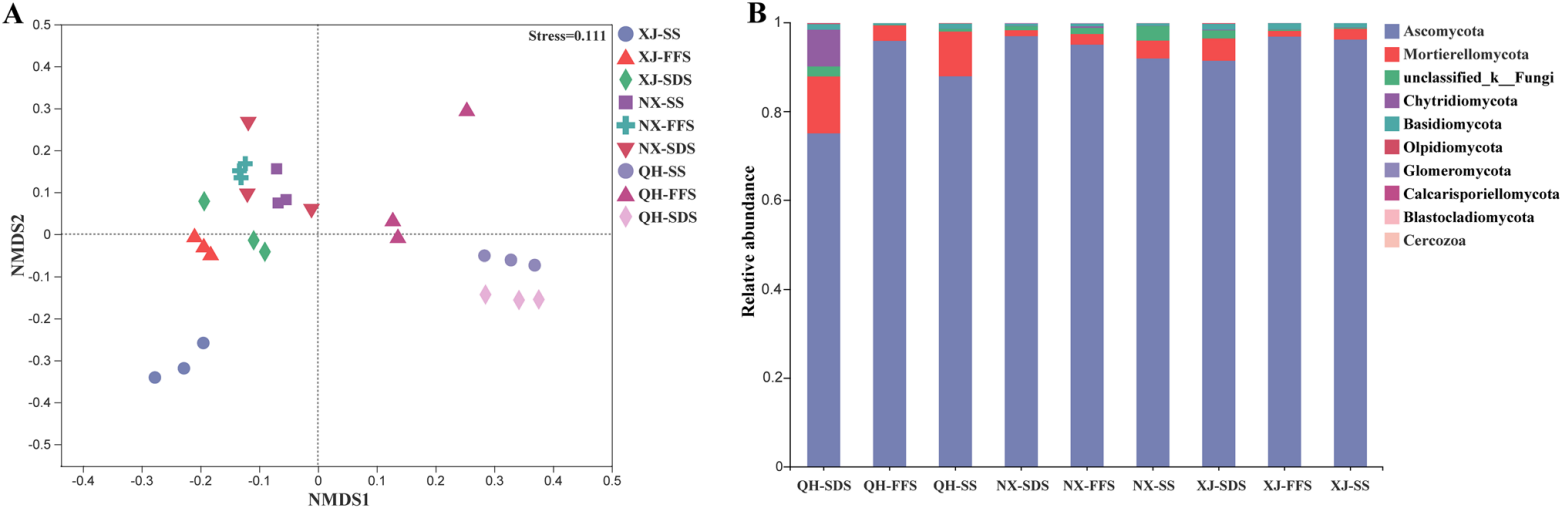
**a** The fungal community composition in the rhizosphere soil of different regions and during different developmental stages. NMDS plots of fungal communities based on Bray–Curtis distances; **b** the relative abundance of soil fungi in each sample at the phylum level

β-Diversity of the samples was analysed based on a Bray–Curtis dissimilarity analysis. A cluster analysis indicated that the samples from the soil resources of the same region were clustered together at the OTU level (Fig. S1B). Then, the variation in the soil fungal communities of different regions was further visualised through nonmetric multiple-dimensional scaling (NMDS) ordination based on the Bray–Curtis distance (Fig. 1A). The results indicated that the NX soil was not clearly separated from the from QH and XJ soil at different developmental stages, which indicated that the development of *L. barbarum* did not significantly alter the community structure. However, the development of *L. barbarum* in QH altered the community structure significantly at different developmental stages.

### Composition of rhizosphere soil fungal communities

Fungal communities were classified into 11 phyla, 29 classes, 172 families, and 332 genera (Table S5). *Ascomycota* and *Mortierellomycota* were the predominant phyla in the rhizosphere fungal communities in all samples (Fig. 1B, Table S6). Interestingly, *Ascomycota* accounted for approximately 92.06–97.08% of the total fungal communities in the NX soil samples (0.92–3.33% were unclassified) and approximately 75.24−96.04% and 91.60−96.97% in the QH and XJ soil samples, respectively. *Mortierellomycota* were significantly enriched in QH soil samples during the SS (13.39%) and SDS (16.70%). *Chytridiomycota* were significantly enriched in the QH soil samples during the SDS (9.98%).

*Sordariomycetes* and *Dothideomycetes* were the predominant classes among the *Ascomycota*, with respective abundances of 70.66–88.92% and 9.72–15.96% in NX, 49.95–53.59% and 5.17–38.56% in QH, and 29.83–89.88% and 4.13–21.59% in XJ during three developmental stages (Fig. S1C, Table S7). The abundance of *Eurotiomycetes* differed significantly across the three regions, with the abundance of 47.34% in XJ during the SS, which was significantly higher than those in samples from NX and QH. The abundance of *Pezizomycetes* (21.6%) was the highest in QH during the SS. The results indicated that the fungal community of the rhizosphere soil in NX is less diverse but highly stable.

### Relationships among fungal communities, soil properties, and active ingredients

**Fig. 2:**
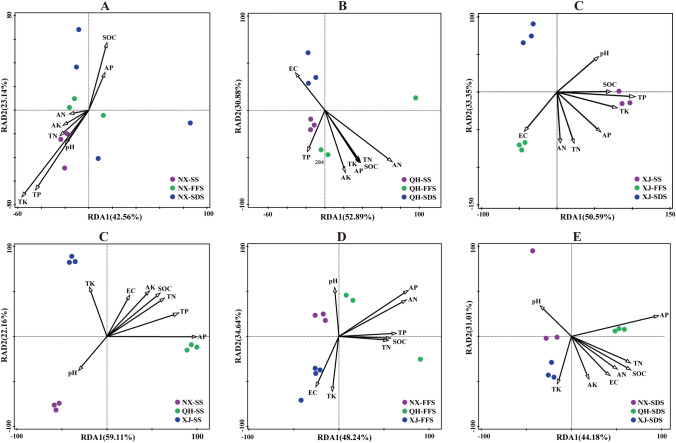
RDA plot depicting the correlation between soil properties and fungal communities in three wolfberry production regions during the three developmental stages. **a**–**c** Samples from Ningxia (NX), Qinghai (QH), and Xinjiang (XJ) in the sprouting stage (SS), flowering and fruiting stage (FFS), and summer dormant stage (SDS); **d**, **e** samples from three wolfberry production regions in the SS, FFS and SDS

**Fig. 3:**
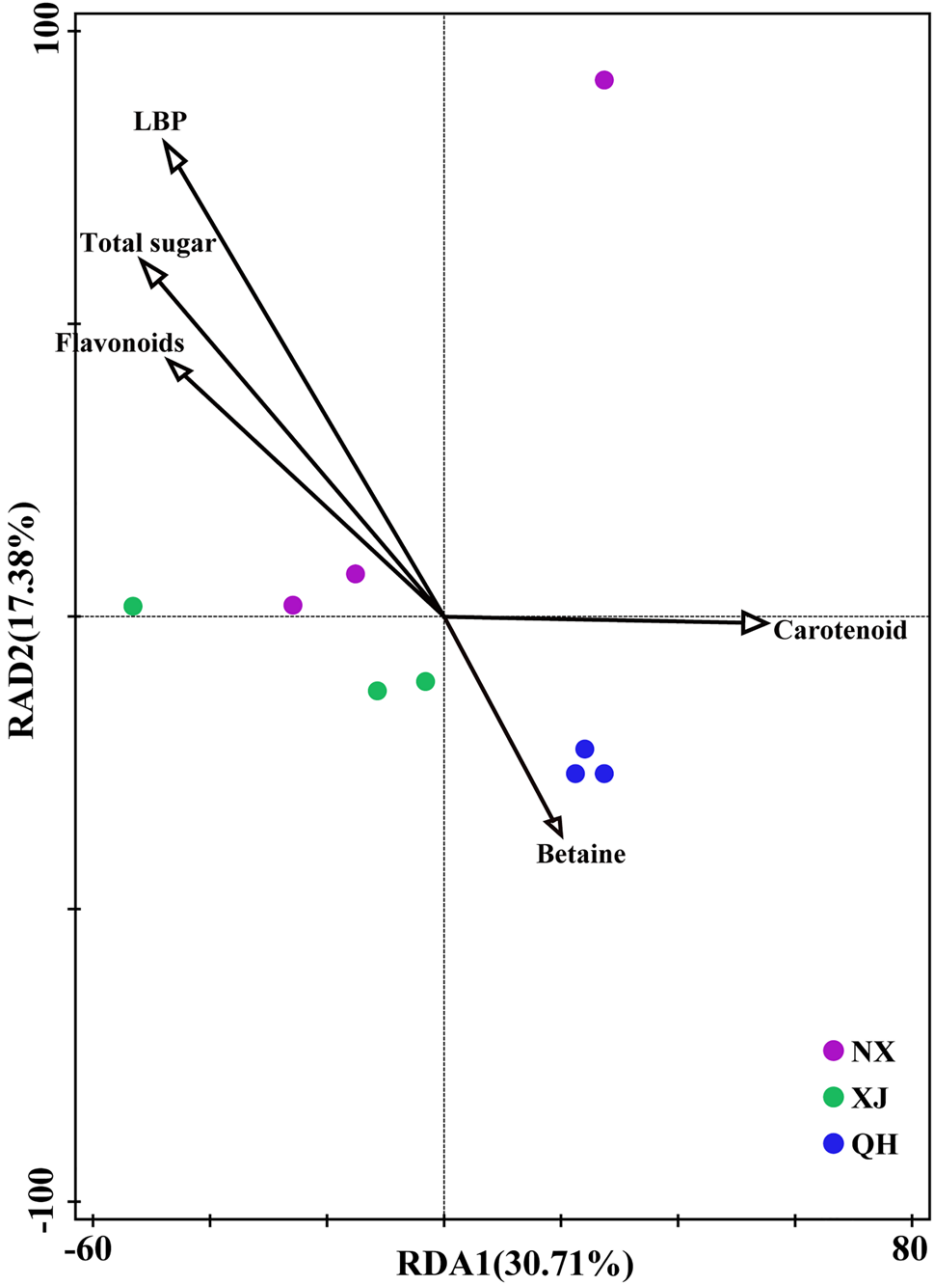
The correspondence analysis for main active ingredients and fungal diversity of rhizosphere soils in three wolfberry production regions

RDA was applied to determine the correlations between soil properties and fungal community composition. The RDA results indicated the strongest correlation of soil TK, AN, and TP with the fungal community structure in different developmental stages of wolfberry in NX, QH and XJ, respectively (Fig. 2A–C). Moreover, soil AP exhibited a significant correlation with the fungal community structure at the same developmental stage in different regions (Fig. 2D, E). These results showed that the main driving factors for the fungal community structure were TK, AN, TP, and AP. Figure 3 illustrates that 48.09% of the variation can be explained by the relationship between fungi and active ingredients. The results showed a significant correlation between LBP and the fungal community composition (Fig. 3).

## Discussion

Soil microorganisms play a pivotal role in the ecosystem and are the key factors associated with soil quality, soil fertility, and productivity. Alterations in the rhizosphere soil microbial community composition affect the absorption and transformation of soil nutrients (Acosta-Martínez 2014). The unique geographical environment and climatic conditions in Ningxia are the major factors that have contributed to the establishment of *L. barbarum* as a well-known geoherb in China, which exhibits a wide range of biological effects, including immuno-modulatory, antiageing, antitumour, neuroprotective, and hepatoprotective effects (Kulczyński and Gramza-Michałowska 2016). However, the effects of the microbial community structure in plant rhizosphere soil on geoherbalism remain to be studied in detail. To efficiently manipulate the microbial populations in the rhizosphere for the benefit of plants, a better understanding of the relative importance of soil and plant factors for microbial rhizosphere communities is required. In the present study, fungal communities associated with the *L. barbarum* rhizosphere soils from different regions were characterised through high-throughput sequencing during the SS, FFS, and SDS.

For detecting species diversity in soil samples, the Illumina HiSeq technology is more efficient and precise than the traditional isolation culture method (Rhodes et al. 2014). In this study, the Illumina HiSeq platform was used to analyse the fungal ITS region, which provided detailed insights into the fungal community patterns in rhizosphere soil of *L. barbarum* in NX, QH, and XJ under three developmental stages. A total of 1,948,798 high-quality reads were detected from the 27 rhizosphere soil samples (Supplementary Table S4). The detection of a large number of effective reads indicated that this high-throughput sequencing technique is suitable for analysing the fungal community composition in rhizosphere soils of *L. barbarum*.

The abundance and variation of the fungal population are crucial for the sustainable development of soil quality, function, and ecosystem (Berg and Smalla 2009). The rhizosphere soil fungal community structure and *L. barbarum* diversity were found to change considerably in the present study. The number of OTUs and alpha diversity index were found to increase continuously with the development of *L. barbarum*. Moreover, the fungal community was more diverse and abundant in NX than in QH and XJ, suggesting that the soil fungal community structure is obviously related to the geoherbalism of *L. barbarum*. Most studies have shown that the growth stage affects the fungal community structure of plant roots. Our results indicated that the NX soil under different developmental stages did not significantly alter the community structure, which may be due to a low number of replicates or greater variances in taxonomic abundances. Soil microbial communities play an essential role in soil nutrient cycling and organic matter dynamics in agro-ecosystems, thereby serving as soil quality indicators. Thus, changes in the soil microbial community composition or total microbial biomass can affect the rhizosphere soil quality (Berg and Smalla 2009). *Ascomycota* and *Mortierellomycota* were found to be the predominant fungal phyla in rhizosphere fungal communities in all samples in the present study. This result is in accordance with our previous results. Moreover, some *Mortierella* species were found to exhibit antagonistic activities against plant pathogens that cause root rot or potato scab. Therefore, *Mortierella* might act as the key factor for soil-borne disease suppression properties of the soil. At the class level, the relative abundances of *Sordariomycetes*, *Dothideomycetes*, *Eurotiomycetes*, and *Pezizomycetes* varied significantly across different samples, which caused differences in the quality of wolfberry across different regions.

The growth rate and metabolic activities of rhizosphere microorganisms are extremely high, which can accelerate the effective decomposition and release of the solidified inorganic mineral elements P and K and the organic mineral elements P and K, as well as promote the availability and absorption of the solidified plant nutrient elements such as P and K in the soil (Marschner et al. 2004; Berg and Smalla 2009). Specific constituents of soil nutrients (e.g., C, N, P and K) and pH may impose physiological constraints on fungal survival and growth, thereby directly altering the fungal community composition (Zhang et al. 2016). According to the RDA results, soil TK, AN and TP had the strongest correlation with the fungal community structure in the developmental process of *L. barbarum.* AP was significantly correlated with the fungal community structure in the three *L. barbarum*-producing areas, indicating that some fungal species effectively convert the insoluble phosphorus and potassium present in the soil into the soluble form, which increases the absorption and utilisation of phosphorus and potassium by plants.

The medicinal value of genuine herbs lies in the type of metabolites, and our results showed a significant correlation of LBPs with the fungal community composition. LBP is one of the main active ingredients of wolfberry. In pharmacological experiments conducted in some studies, LBP of wolfberry has been shown to exert antiageing, antihyperlipidemia, obesity-improving, antifatigue, antitumour, antioxidation, and BMD-increasing effects (Jin et al. 2013; Yu et al. 2020a). We also found a positive correlation of the *Trichocomaceae*, *Chaetomiaceae*, and *Sporormiaceae* abundances with the LBP content at the family level. Thus, investigations of the fungal community in rhizosphere soil could provide new insights into the mechanism of geoherb formation in NX wolfberry.

## Supplementary Information

Below is the link to the electronic supplementary material.

Supplementary file 1 **Fig. S1** a. Rarefaction curves for each sample; b. Cluster analysis of different samples based on OTUs; c. The relative abundance of soil fungi in each sample at the family level (TIF 1343 KB)

Supplementary file 2 **Table S1** Geographical factors for different sampling positions. (XLS 12 KB)

Supplementary file 3 **Table S2** Basic physicochemical properties of rhizosphere soils at different developmental stages of *Lycium barbarum* L. (XLS 20 KB)

Supplementary file 4 **Table S3** Content analysis of the main active ingredients of *Lycium barbarum* L. (XLS 12 KB)

Supplementary file 5 **Table S4** Statistics and details of the sequencing data. (XLS 12 KB)

Supplementary file 6 **Table S5** The information of OTUs in all the samples. (XLS 283 KB)

Supplementary file 7 **Table S6** Relative abundance of fungal phyla in rhizosphere soil samples. (XLS 40 KB)

Supplementary file 8 **Table S7** Relative abundance of fungal family in rhizosphere soil samples. (XLS 34 KB)
