# Supplementary figures and images for: Diversity and spatiotemporal dynamics of fungal communities in the rhizosphere soil of Lycium barbarum L.: a new insight into the mechanism of geoherb formation
Source: Arch Microbiol. 2022 Feb 26;204(3):197. doi: 10.1007/s00203-022-02781-5 (PMC8881256; doi:10.1007/s00203-022-02781-5)

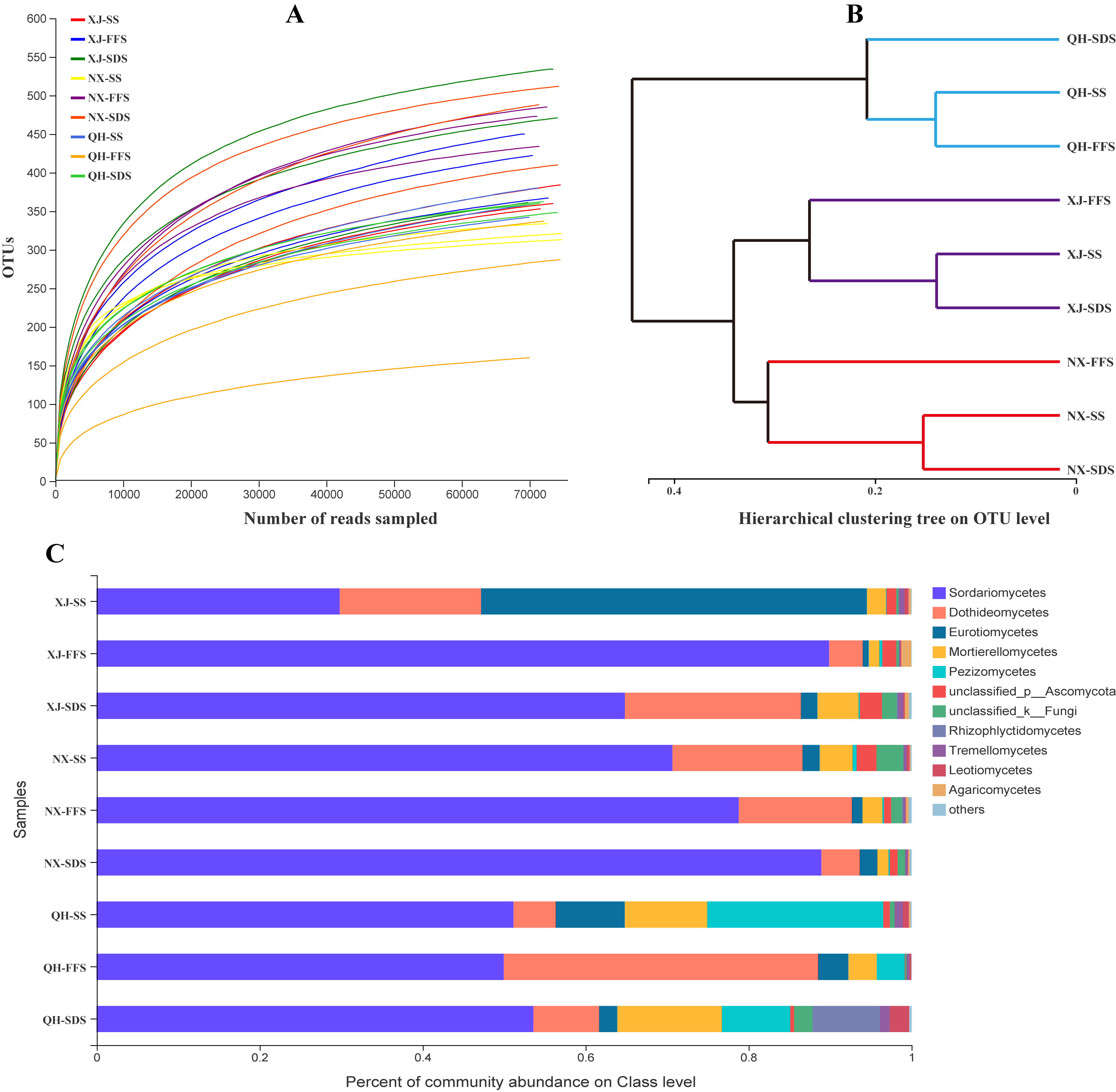

Supplement: Supplementary file 1 — Fig. S1 a. Rarefaction curves for each sample; b. Cluster analysis of different samples based on OTUs; c. The relative abundance of soil fungi in each sample at the family level (TIF 1343 KB) [file 203_2022_2781_MOESM1_ESM.tif]
